# Adolescent Intermittent Ethanol Exposure Effects on Kappa Opioid Receptor Mediated Dopamine Transmission: Sex and Age of Exposure Matter

**DOI:** 10.3390/brainsci10080472

**Published:** 2020-07-23

**Authors:** Mary B. Spodnick, Raymond T. Amirault, Trevor T. Towner, Elena I. Varlinskaya, Linda P. Spear, Anushree N. Karkhanis

**Affiliations:** Developmental Exposure Alcohol Research Center, Center for Developmental and Behavioral Neuroscience, Department of Psychology, Binghamton University-SUNY, Binghamton, NY 13902, USA; mspodni1@binghamton.edu (M.B.S.); ramirau1@bimghamton.edu (R.T.A.); ttowner1@binghamton.edu (T.T.T.); varlinsk@binghamton.edu (E.I.V.); lspear@binghamton.edu (L.P.S.)

**Keywords:** adolescent intermittent ethanol exposure, dopamine, kappa opioid receptors, nucleus accumbens

## Abstract

Underage alcohol drinking increases the risk of developing alcohol use disorder (AUD). In rodents, adolescent ethanol exposure augments ethanol consumption and anxiety-like behavior while reducing social interaction. However, the underlying mechanisms driving these adaptations are unclear. The dopamine and kappa opioid receptor (KOR) systems in the nucleus accumbens (NAc) are implicated in affective disorders, including AUD, with studies showing augmented KOR function and reduced dopamine transmission in ethanol-dependent adult animals. Thus, here we examine the impact of adolescent intermittent ethanol (AIE) exposure on dopamine transmission and KOR function in the NAc. Rats were exposed to water or ethanol (4 g/kg, intragastrically) every other day during early (postnatal day (PD) 25–45) or late (PD 45–65) adolescence. While AIE exposure during early adolescence (early-AIE) did not alter dopamine release in male and female rats, AIE exposure during late adolescence (late-AIE) resulted in greater dopamine release in males and lower dopamine release in females. To determine the impact of AIE on KOR function, we measured the effect of KOR activation using U50,488 (0.01–1.00 µM) on dopamine release. Early-AIE exposure potentiated KOR-mediated inhibition of dopamine release in females, while late-AIE exposure attenuated this effect in males. Interestingly, no differences in KOR function were observed in early-AIE exposed males and late-AIE exposed females. Together, these data suggest that AIE exposure impact on neural processes is dependent on sex and exposure timing. These differences likely arise from differential developmental timing in males and females. This is the first study to show changes in KOR function following AIE exposure.

## 1. Introduction

Adolescence is a key period for the initiation of alcohol use [1] and the beginning of the development of alcohol use disorder (AUD) [2]. Approximately 58% of teenagers report having at least one alcoholic drink prior to reaching 18 years of age [3]. Moreover, about 4.7% of adolescents (ages 12–17) and about 35% of young adults (ages 18–25) have engaged in binge drinking in the past month [3]. Initiation of binge drinking patterns before the age of 14 is especially concerning, given that the prevalence of alcohol dependence following early initiation (before 14 years) is four times greater than if individuals wait until the legal age limit [4,5,6,7]. Earlier age of initiation of alcohol consumption is also correlated with mental health issues, including AUD, later in life [2,8,9].

Similar to humans, adolescent ethanol exposure in rodents promotes long-lasting maladaptive behaviors [10], including augmented, anxiety-like behavior [11,12] and attenuation of social investigation and preference [13]. These changes are differentially affected by the age of exposure in males and females; specifically, while both early adolescent (postnatal day (PD) 25–45) and late adolescent (PD 45–65) exposures result in greater anxiety-like behavior in males, only early exposure females exhibit augmented anxiety-like behavior [12]. With respect to social interaction, social investigation and social preference were significantly reduced in early-exposed males, with no social alterations evident in late-exposed males and females regardless of exposure timing [13]. Social interaction has a substantial reward value, with mesolimbic (ventral tegmental area (VTA) to nucleus accumbens (NAc)) dopamine projections being directly involved in the modulation of social rewards [14,15]. Thus, it is possible that adolescent alcohol exposure-associated social deficits and enhanced anxiety-like behaviors may be driven by neurochemical changes in the NAc, particularly since early–mid adolescence is a critical time for maturation of mesolimbic dopamine neurons [16,17].

Rodent studies have demonstrated a direct relationship between ethanol and dopamine, with acute ethanol challenge resulting in the activation of dopamine neurons in the VTA [18,19], subsequently elevating extracellular dopamine levels in the NAc core of adult male rats [20]. In contrast, chronic intermittent ethanol (CIE) exposure in adult male mice attenuates stimulated dopamine release during acute withdrawal in the NAc core [21,22]. These CIE-mediated reductions in dopamine have been shown to last for up to 72 h after the cessation of final exposure [21,22,23]. Relatively fewer studies, however, have explored ethanol’s impact on dopamine transmission following adolescent exposure. There is some evidence that adolescent ethanol exposure between postnatal days (PDs) 30 and 45 can decrease accumbal dopamine tissue content in male mice when assessed following five days of abstinence [24]. Interestingly, intermittent ethanol exposure between PDs 30 and 50, followed by 15 days of abstinence, resulted in elevated basal levels of dopamine in the NAc in adult rats [25]. On the other hand, a protracted abstinence of 25 days following intragastric, intermittent ethanol exposure during early adolescence (PD 25–45) revealed no differences in stimulated dopamine release at baseline in the NAc core of adult rats [26]. Although there is some overlap in the age of exposure across these studies, a period of abstinence and the five-day shift in exposure time may drive the differences observed, as maturation of the dopamine system is at its peak during this time [16,17]. The focus of this study, accordingly, is to systematically compare the impact of ethanol exposure during early–mid adolescence and late adolescence/emerging adulthood [27,28]. These age spans correspond to approximately 10–18 and 18–25 years of age in humans, respectively [28].

Among the various mechanisms that regulate dopamine release in the NAc [29], the inhibitory control of kappa opioid receptors (KORs) is of particular importance, given that the NAc is also a target of ethanol. Specifically, KORs are located on the terminals of accumbal dopamine neurons [30], and activation of these receptors inhibits dopamine release [21,22,31,32,33] and reduces the extraction fraction of dopamine, which an indirect measure of dopamine uptake [34]. This KOR-mediated inhibition of dopamine release in the NAc core is enhanced in alcohol-dependent adult male mice [21,22]; a systemic KOR blockade, in turn, reverses dependence-induced enhancement in ethanol consumption in mice [21] and rats [35]. Conversely, adult male rats with moderate drinking history show increased ethanol intake in response to KOR inhibition [36]. Adding to the complexity of this picture is that adult female rats with moderate drinking history show reduced ethanol intake following a KOR blockade, whereas KOR manipulation does not affect ethanol intake in adolescent male and female rats [36]. Given the relationship between KORs and dopamine illustrated by these alcohol exposure models, it is possible that adolescent intermittent ethanol (AIE) exposure may lead to sex- and age-dependent disruptions in dopamine transmission via changes in KOR function.

While there is a paucity in the literature examining AIE-mediated neurobiological changes, studies measuring affect-related behavioral effects show that AIE-induced behavioral adaptations are dependent on time of exposure—either early adolescence (PD 25–45) or late adolescence/emerging adulthood (PD 45–65)—and sex [12,13]. Thus, in this study we examined the impact of AIE exposure during early and late adolescence, followed by forced, protracted abstinence in male and female rats, on dopamine transmission and KOR function, using ex vivo fast-scan cyclic voltammetry (FSCV). Based on previous behavioral [12,13] and neurobiological [26] data, we hypothesized that AIE exposure during early adolescence will not alter dopamine efflux at baseline; however, late adolescent exposure will only attenuate dopamine release in males. Moreover, we predicted that KOR function will be potentiated following AIE exposure during early adolescence in both sexes, but only in males exposed to AIE during late adolescence. This is the first study to examine the effects of AIE exposure on KOR function in the NAc.

## 2. Methods

### 2.1. Experimental Subjects

Male and female Sprague Dawley rats derived from our breeding colony at Binghamton University were used. On PD 1, litters were preferentially culled to a ratio of six males and four females when possible. On PD 21, rats were weaned and housed in pairs with a same-sex littermate. In order to limit the possible confounding of litter effect [37], one rat per sex per litter was assigned to each experimental group. Rats were maintained on a 12:12 h light/dark schedule (lights on at 700 h) with standard rat chow (LabDiet 5L0D–PicoLab Laboratory Rodent Diet, ScottPharma Solutions, Marlborough, MA, USA), and tap water available ad libitum. Maintenance and experimental treatment of rats was in accordance with the National Institutes of Health guidelines for animal care, using procedures approved by the Binghamton University Institutional Animal Care and Use Committee(IACUC protocol number: 818-19).

### 2.2. Adolescent Intermittent Ethanol Exposure

**Figure 1:**
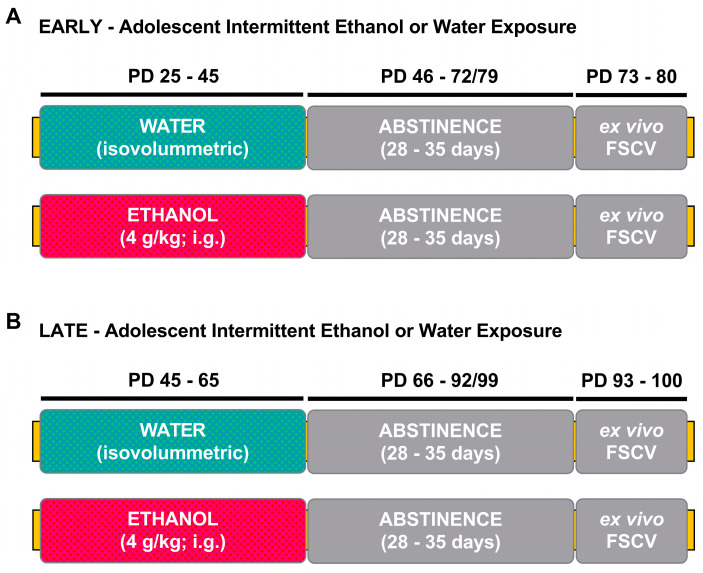
Timeline of experimental procedure. (**A**) Experimental manipulation during early adolescence. Male and female Sprague–Dawley rats were exposed intermittently every other day, for a total of 11 exposures, to water or ethanol (early AIE) via intragastric gavage between PDs 25 and 45. After PD 45, all rats were subject to a period of protracted abstinence for 28–35 days, during which no manipulation took place. Ex vivo FSCV was conducted during adulthood, between PD 73 and 80. (**B**) Experimental manipulation during late adolescence. During PD 45–65, male and female rats were exposed intermittently every other day, for a total of 11 exposures, to either water or ethanol (late AIE) via intragastric gavage. After PD 65, all rats were subject to protracted abstinence for 28–35 days, during which no manipulation took place. Ex vivo FSCV was conducted during adulthood (between PD 93–100). AIE: adolescent intermittent ethanol; PD: postnatal day; FSCV: fast-scan cyclic voltammetry.

Rats were exposed to ethanol (4 g/kg, 25% *v*/*v*) via intragastric gavage (i.g.) during early/mid (early AIE, PD 25–45) or late (late AIE, PD 45–65) adolescence. Age- and sex-matched controls were administered via an isovolumetric amount of water. Animals were given ethanol or water every other day during the light phase, between 1300–1600. An intermittent pattern of exposure was used to better represent human alcohol consumption [38]. Experimental timelines can be seen in Figure 1A (early-AIE exposure) and Figure 1B (late-AIE exposure).

We used ex vivo FSCV to determine AIE-induced changes in dopamine kinetics and KOR function within the NAc core. All ex vivo FSCV recordings were conducted in adulthood after 28–35 days of forced abstinence (PD 73–80 for early-AIE exposure and PD 93–100 for late-AIE exposure). Ex vivo FSCV procedures were similar to those used in previous studies [32]. Briefly, rats were anesthetized with isoflurane and euthanized via rapid decapitation. Brains were collected promptly and immersed in cold, oxygenated, artificial cerebrospinal fluid (aCSF), and then sliced using a vibratome (VT1200 S, Leica BioSystems, Buffalo Grove, IL, USA). Brain slices 400 µm thick containing the NAc core [39] were obtained and transferred to a recording chamber with a continuous flow of oxygenated aCSF (32 °C)—one slice per rat was used for recordings. Dopamine efflux was induced using a bipolar stimulating electrode (8IMS3033SPCE, Plastics One, Roanoke, VA, USA) and measured using a carbon fiber recording electrode (~150 µm length, 7 µM radius; C 005722/6, Goodfellow, UK). The recording and stimulating electrodes were placed approximately 100 µm apart. The stimulating electrode was placed on the slice’s surface, while the recording electrode was placed approximately 100 µm below the surface of the slice. Dopamine efflux was induced through the use of a single, rectangular, 4.0 ms duration electrical pulse (350 µA, monophasic, inter-stimulus interval: 300 s). To detect dopamine release, a triangular waveform (−0.4 to +1.2 to −0.4 V vs. silver/silver chloride, 400 V/s) was applied every 100 ms to the recording electrode. Baseline dopamine recordings were taken prior to the bath application of any drug. Once stable baseline recordings were obtained, the KOR agonist U50,488 was bath applied in cumulative concentrations (0, 0.01, 0.03, 0.1, 0.3, and 1.0 µM) to determine AIE-associated changes to KOR-mediated dopamine release. Recording electrodes used for each experiment were calibrated using a 3.0 µM concentration of dopamine, in order to quantify the measured dopamine release and dopamine kinetics. FSCV recordings were later analyzed using Demon Voltammetry and Analysis software [40]. Dopamine kinetics were determined through modeling the stabilized signals using Michaelis–Menten kinetics [41].

### 2.3. Statistical Analysis

All data in this study were analyzed using GraphPad Prism 8 (GraphPad Software, La Jolla, CA, USA). Because our main focus was on examining the impact of AIE exposure on dopamine kinetics in males and females, and because the animals exposed in early and late adolescence were tested at different ages in adulthood, we analyzed the data separately for the early and late exposure groups. For each age of exposure, the dependent variables’ dopamine release and reuptake rate were analyzed, using separate analysis for each measure and using a 2 (adolescent exposure: water, AIE) × 2 (sex) analyses of variance (ANOVA). Percent baseline dopamine release in response to a selective kappa agonist U50,488 was analyzed separately for each sex. Using a 2 (adolescent exposure: water, AIE) × 5 (U50,488 concentration: 0.01, 0.03, 0.1, 1.0, 3.0 µM) repeated measures ANOVA, with the U50,488 concentration treated as a repeated measure. All main effects and interactions were explored using Holm–Sidak’s pairwise post hoc analyses. Non-linear regression analysis was used to calculate the effective concentration of the drug causing a half-maximal effect (EC50), in order to examine shifts in potency. Maximal change in dopamine release was calculated to examine changes in efficacy. Student’s *t*-tests were used to determine AIE exposure-induced changes in potency and efficacy within each age of exposure/sex condition. All data are reported as mean ± standard error of the mean. The significance level for all statistical measures was set at *p* < 0.05.

## 3. Results

### 3.1. Late-AIE Exposure Disrupts Dopamine Release in Male and Female Rats

**Figure 2:**
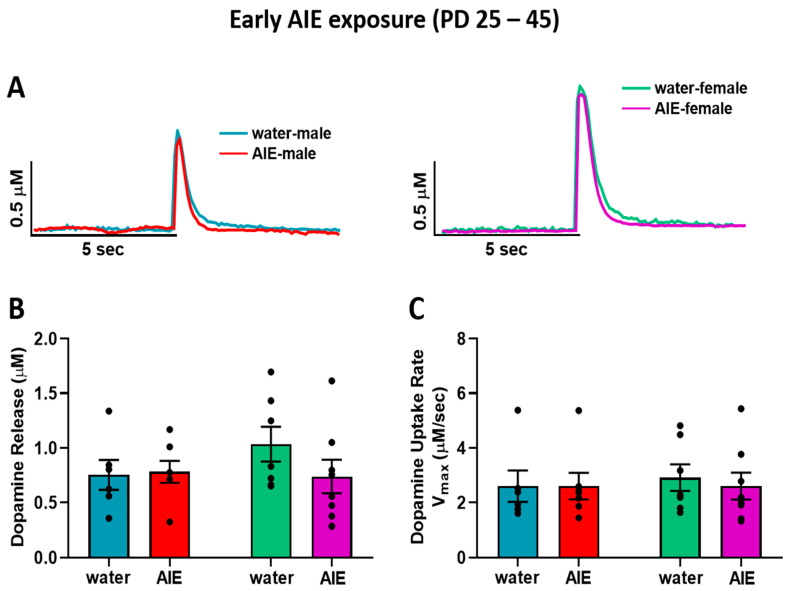
Baseline dopamine kinetics in the NAc core after early adolescent exposure (PD 25–45). (**A**) Representative transient dopamine signals in response to a single-pulse electrical stimulation, indicating concentration of dopamine released over time from the NAc core of one rat in each early exposure group (left: water-exposed male = blue, AIE- exposed male = red; right: water-exposed female = green, AIE-exposed female = purple). (**B**) Early AIE exposure had no effect on evoked dopamine release in male and female rats. (**C**) Similarly, the uptake rate of dopamine was not altered following early AIE exposure in both males and females. NAc: nucleus accumbens; AIE: adolescent intermittent ethanol.

In order to identify the impact of AIE exposure on dopamine kinetics in the NAc core, we measured electrically stimulated dopamine release and reuptake rate in adult male and female rats exposed to AIE or water during early or late adolescence, using ex vivo FSCV. We assessed the early (male: water = 6, AIE = 7; female: water = 7, AIE = 8) and late (male: water = 6, AIE = 6; female: water = 6, AIE = 8) exposure groups separately. Figure 2A shows representative traces of transient accumbal dopamine signals in response to a single pulse electrical stimulation in adult male (left) and adult female (right) rats exposed to water or ethanol during early adolescence. Baseline dopamine release (Figure 2B) and rate of uptake (Figure 2C) did not differ as a function of early adolescent exposure in adult males and females.

**Figure 3:**
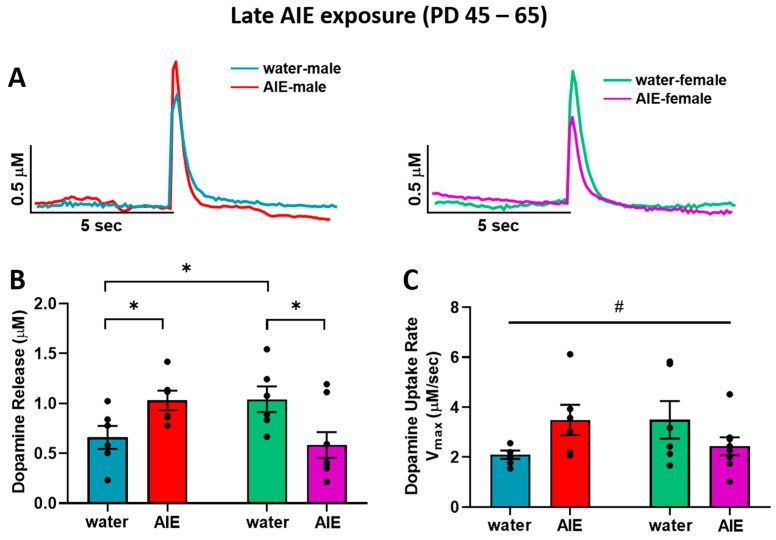
Baseline dopamine kinetics in the NAc core after late adolescent exposure (PD 45–65). (**A**) Representative transient dopamine signals in response to a single-pulse electrical stimulation, indicating concentration of dopamine released over time from the NAc core of one rat in each late exposure group (left: water-exposed male = blue, AIE-exposed male = red; right: water-exposed female = green, AIE-exposed female = purple). (**B**) Dopamine release in water-exposed females was significantly greater than dopamine release in water-exposed males. In males, late AIE-exposure (red bar) led to greater stimulated dopamine release compared to water exposed controls (blue bar). In females, late AIE-exposed rats (purple bar) exhibited lower stimulated dopamine release compared to water-exposed controls (green). (**C**) Analysis of the impact of AIE exposure on dopamine uptake rate revealed an interaction between sex and exposure. NAc: nucleus accumbens; AIE: adolescent intermittent ethanol. * A significant difference between water and AIE-exposed rats within the same sex or between sexes in water-exposed groups where *p* < 0.05; # sex by treatment interaction where *p* < 0.05.

Figure 3A shows representative traces of evoked dopamine response in the NAc core of adult male (left) and female (right) rats exposed to water and AIE during late adolescence. In contrast to early adolescent exposure, AIE exposure during late adolescence resulted in altered dopamine release (Figure 3B). A two-way ANOVA of dopamine release revealed an interaction between late adolescent exposure (water vs. AIE) and sex (*F*_(1,22)_ = 10.5; *p* < 0.01), with late AIE exposure resulting in significantly lower dopamine release relative to water-exposed controls in females, but leading to elevated dopamine release in males compared to water-exposed controls (*p* < 0.05). In addition, water-exposed females showed significantly greater dopamine release compared to water-exposed males (*p* < 0.05). Although the ANOVA of dopamine uptake rate revealed a significant exposure by sex interaction (Figure 3C; *F*_(1,22)_ = 5.85; *p* < 0.05), post hoc analyses did not identify any significant differences between water and AIE-exposed males and females. Collectively, the effects of AIE on dopamine release in the NAc core appear to be impacted by exposure timing and sex.

### 3.2. AIE Exposure Affects Accumbal KOR Function in an Age- and Sex-Dependent Manner

**Figure 4:**
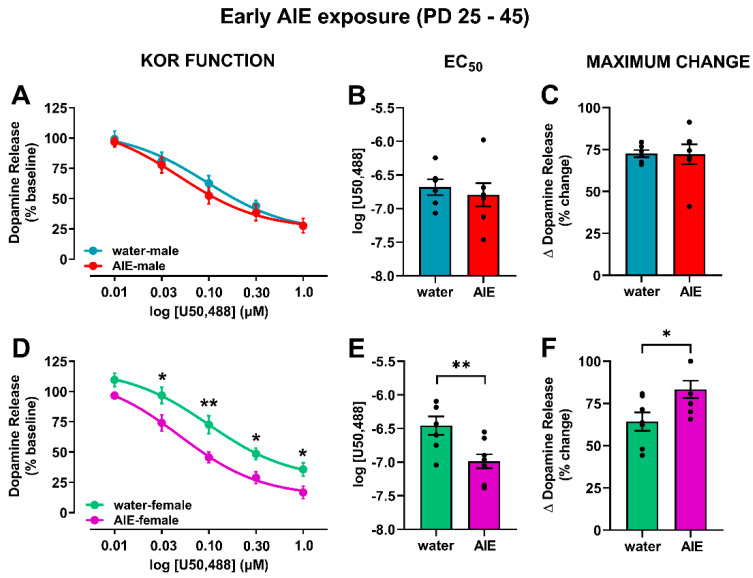
Impact of early AIE exposure on KOR function and its regulation of dopamine release. The KOR agonist U50,488 was bath-applied to the tissue in cumulative concentrations, and electrically stimulated dopamine release was recorded at each concentration in male (**A**–**C**) and female (**D**,**E**) rats. (**A**) In males, cumulative concentrations of U50,488 decreased dopamine release regardless of adolescent exposure to water or ethanol. (**B**) No differences in the EC50 for U50,488 were evident between water- and AIE-exposed exposed males. (**C**) Similarly, no differences in the maximal change in dopamine release were found between water- and AIE-exposed male rats. (**D**) In females, both water- and AIE-exposed animals showed KOR activation-mediated inhibition of dopamine release; however, this effect was enhanced in AIE relative to water-exposed rats at 0.03, 0.10, 0.30, and 1.00 µM of U50,488. These data suggest that early AIE-exposure resulted in augmented KOR function. Early AIE exposure-associated potentiation of KOR function was driven by increased potency, as shown by the reduced EC50 of U50,488 (**E**), as well as increased efficacy, as shown by significantly greater maximum change in dopamine release (**F**) in early AIE-exposed females compared to water-exposed controls. There was a significant difference between water- and AIE-exposed rats within sex: * *p* < 0.05; ** *p* < 0.01. KOR: kappa opioid receptor; NAc: nucleus accumbens; AIE: adolescent intermittent ethanol; EC50: effective concentration of the drug causing a half-maximal effect.

In order to determine the effects of AIE exposure on KOR function in the NAc core, we measured the functional responsiveness of KORs in water- and AIE-exposed male and female rats by measuring dopamine release following bath application of cumulative concentrations of a selective KOR agonist, U50,488 (0.01–1.0 µM), to accumbal slices. To further assess if AIE exposure affected potency and efficacy, we calculated the EC50 of U50,488 and the maximum effect of KOR activation on dopamine inhibition, respectively. In early-exposure males (Figure 4A), a main effect of U50,488 concentration (*F*_(4,44)_ = 142.8; *p* < 0.0001) was observed, with no significant difference between early AIE-exposed and control groups. Likewise, no differences between early AIE-exposed and water-exposed males were observed in the EC50 (Figure 4B) and maximum effect (Figure 4C) of U50,488. In contrast, AIE exposure during early adolescence augmented KOR function in female rats relative to the water-exposed control group (Figure 4D). The ANOVA revealed main effects of early adolescent exposure (water vs. AIE; *F*_(1,13)_ = 10.4; *p* < 0.01) and U50,488 concentration (*F*_(4,52)_ = 157.3; *p* < 0.0001). U50,488 dose-dependently decreased dopamine release, and early AIE-exposed females demonstrated more pronounced decreases than their water-exposed counterparts, especially at 0.03, 0.10, 0.30, and 1.00 µM of U50,488 (*p* < 0.05), suggesting augmented KOR function in response to the agonist mediation activation. This hyperfunction of KORs was driven by an increase in potency (Figure 4E; *t*_13_ = 3.14; *p* < 0.01) and efficacy (Figure 4F; *t*_13_ = 2.55; *p* < 0.05). These data suggest that the consequences of AIE exposure during early adolescence, followed by protracted abstinence, differ between males and females; specifically, AIE exposure increases KOR function selectively in female rats.

**Figure 5:**
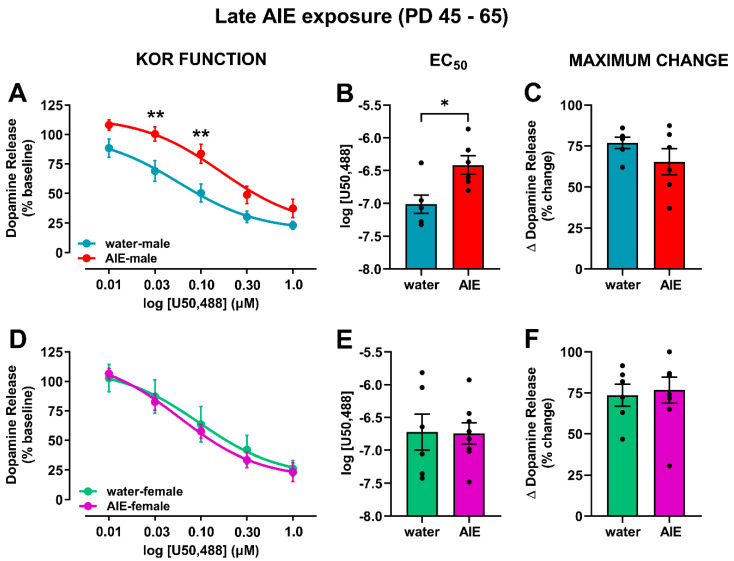
Impact of late AIE-exposure on KOR function and its regulation of dopamine release. The KOR agonist U50,488 was bath-applied to the tissue in cumulative concentrations, and electrically stimulated dopamine release was recorded at each concentration in male (**A**–**C**) and female (**D**,**E**) rats. (**A**) In males, U50,488 concentration dependently inhibited stimulated dopamine release in both AIE and water exposed rats; however, this effect was significantly attenuated in rats exposed to AIE, particularly at 0.03 and 0.1 µM of U50,488. These data suggest that late AIE-exposure reduced KOR function in male rats. (**B**) The AIE-associated attenuation in KOR function was driven by reduced potency, as shown by the augmented EC50 of U50,488 in late-AIE-exposed male rats compared to water-exposed controls. (**C**) No differences were observed in the maximal effect of U50,488 on dopamine release between the AIE- and water-exposed groups, indicating that efficacy did not drive the changes in KOR function. (**D**) In females, bath application of U50,488 decreased stimulated dopamine release in a similar manner for both water- and AIE-exposed rats. No differences in potency (**E**) and efficacy (**F**) were observed across the two groups. There was a significant difference between water and AIE-exposed rats within sex: * *p* < 0.05; ** *p* < 0.01; KOR: kappa opioid receptor; NAc: nucleus accumbens; AIE: adolescent intermittent ethanol; EC50: effective concentration of drug causing a half-maximal effect.

Contrary to the early-AIE exposure, late-AIE exposure followed by protracted abstinence attenuated KOR function in male rats, with no change observed in females (Figure 5). In males, the ANOVA comparing KOR activation-mediated inhibition of dopamine release (Figure 5A) revealed the main effects of exposure (water vs. AIE; *F*_(1,10)_ = 10.8; *p* < 0.01) and U50,488 concentration (*F*_(4,40)_ = 63.7; *p* < 0.0001). As expected, U50,488 concentration dependently decreased dopamine release in both groups; however, late-AIE-exposed males showed less pronounced decreases than their water-exposed counterparts, suggesting reduced functional response of the KORs to the U50,488-mediated activation in late-AIE males. Post hoc comparisons showed a significant difference at the 0.03 µM and 0.10 µM concentrations of U50,488 (*p* < 0.01). This hypofunction of KORs was driven by a shift in potency (Figure 5B; *t*_10_ = 3.00; *p* < 0.05), but no difference in efficacy (Figure 5C; *t*_10_ = 1.35; *p* = 0.21). In females, the ANOVA comparing KOR-mediated inhibition of dopamine release showed a main effect of U50,488 concentration (Figure 5D; *F*_(4,48)_ = 107.5; *p* < 0.0001). However, AIE exposure during late adolescence in female rats did not result in altered KOR function in adulthood. As expected, no difference in efficacy and potency were observed between water- and AIE-exposed rats (Figure 5E,F).

## 4. Discussion

In the current study, our data showed that AIE exposure-induced changes in dopamine transmission and KOR function were dependent on age of exposure as well as sex. We observed a sexually dimorphic effect of late AIE exposure on baseline dopamine release: while stimulated dopamine release was greater in AIE-exposed males, it was lower in AIE-exposed females when compared to their respective control counterparts. These differences were not observed in early-exposure rats. In addition, neither early nor late AIE exposure influenced the rate of dopamine uptake in the NAc. With regard to KOR function, our data showed a reduction in KOR function in late AIE-exposed males and an augmentation of KOR function in early AIE-exposed females, when compared to their respective water-exposed controls. We did not observe any changes in KOR function in late AIE-exposed females or early AIE-exposed males. Overall, these data suggest that the long-lasting impact of AIE on dopamine transmission and KOR function is dependent upon age of exposure and sex.

Adolescence is a critical period for maturation of the dopamine system [17]. Dopamine neurons in the VTA exhibit a greater firing rate in adolescent compared to adult rats [16,17]. Dopamine neuron firing increases from birth to early- and mid-adolescence (PD 42–48: period of peak dopamine firing rate), followed by a decline in activity until adulthood (>PD 70), forming an inverted U-curve in male rats [17,42,43,44,45,46]. The greater activity in adolescence seems to be driven by an elevated ratio of phosphorylated tyrosine hydroxylase (TH) levels over total TH in midbrain tissue in adolescents vs. adults [16]. It is possible that in male rats, ethanol exposure during late adolescence interferes with the typical pruning process, resulting in a maintenance of elevated, adolescent-like dopamine neuron activity, as shown by greater stimulated dopamine release in late-AIE-exposed versus water-exposed rats in the current study. The interference in the pruning process may be a result of intermittent ethanol-mediated excitation of these developing neurons during every ethanol exposure, i.e., the excitation surges caused by each ethanol exposure may result in potentiated dopamine release at baseline. Data in the current study showing increased dopamine release in late-AIE exposed males are congruent with a previous study showing elevated levels of dopamine in male rats exposed to ethanol from PD 30 to 50 [25]. These changes are likely not modulated by dopamine uptake, as there are no AIE-induced persistent changes in the rate of dopamine uptake in adulthood [25]. In addition, it is important to note that the effects of ethanol on dopamine regulation primarily occur through its actions on release, and not uptake [47]. Because the dopamine system is in a pre-pruning/growing state during early-mid adolescence, the ethanol-mediated excitation of dopamine neurons likely has minimal maladaptive changes in the dopamine system, further leading to “normal” baseline release in adulthood. Notably, data from the current study are congruent with a study that implemented in vivo FSCV and likewise found no change in baseline dopamine release in early-AIE-exposed male rats following a 25-day forced abstinence period [26].

The development of the dopamine system has been studied relatively less in females. Developmental studies discussed in this paragraph were conducted on females only. Expression of Nurr1, a genetic transcription factor that activates the transcription of dopamine and the vesicular monoamine transporters, increases between PD 28 and 60 in female rats [48]. Furthermore, in adulthood, Nurr1 is crucial in the maintenance and survival of dopamine neurons [49,50]. On the other hand, expression of Pitx3, a transcription factor observed in mesolimbic dopamine neurons and involved in activating the transcription of TH, was observed to steadily decline with age (PD 7–60) [48]. Similarly, TH immunoreactivity also showed an age-related decline throughout early adolescence and into young adulthood [48]. These data suggest that maturation and pruning of the dopamine system likely occurs much earlier in females compared to maturation in males, as described earlier. Therefore, it is possible that the late adolescent female neurochemistry is comparable to adult male neurochemistry, resulting in similar chronic ethanol exposure-mediated changes to the dopamine system. CIE-exposed male mice exhibit lower stimulated dopamine release at baseline compared to controls, up to 72 h post-cessation of the last exposure [21,22]. These data may explain the reduced dopamine levels in late AIE-exposed females in the current study. Because early AIE exposure ends during the developmental phase, the system may compensate and recover “normal” dopamine release. However, late AIE exposure ends towards the end of the developmental phase, likely arresting the dopamine system in an altered state. While acute ethanol potentiates dopamine neuron firing in the VTA [19] and elevates extracellular levels of dopamine in the NAc shell and core [20,51] in adult male rodents, repeated cycles of ethanol exposure and withdrawal result in metabolic tolerance in adolescent mice [52] and tolerance of dopamine response to subsequent acute ethanol exposure during acute withdrawal in adult mice [22]. The withdrawal phase also diminishes dopamine system responsivity to salient stimuli, such as an electrical stimulation [21,22]. It is possible that repeated excitation of the mesolimbic dopamine neurons, resulting in enhanced dopamine release in projection areas, drives the system to overcompensate, leading to an overall hypodopamine state, as observed in the late-AIE-exposed female rats in the current study. It is important to note, however, that basal dopamine levels in the VTA in moderate drinking, adult, ethanol-deprived, alcohol-preferring females is not different from adult, ethanol-naïve, alcohol-preferring females [53]. Future experiments should look at the impact of these AIE exposures during acute withdrawal to test these hypotheses.

As mentioned earlier, KORs on dopamine neuron terminals have inhibitory control over dopamine release. Many studies examining the impact of chronic ethanol exposure in adult animals show ethanol exposure-mediated augmentation in KOR function. Specifically, studies demonstrate greater KOR-mediated inhibition of dopamine release in CIE-exposed adult male mice [21,22]. Moreover, inhibition of KORs attenuates ethanol consumption selectively in dependent animals [21,54]. This interaction between KORs and dopamine prompted us to determine whether the baseline changes we observed in late AIE-exposed rats were driven by AIE-associated changes in KOR function and their control over dopamine release. Interestingly, KOR-mediated inhibition of dopamine release was significantly lower in late AIE-exposed males relative to water-exposed controls, suggesting an attenuation in KOR function. This attenuation potentially explains the greater stimulated dopamine in late AIE-exposed exposed males relative to their water-exposed counterparts. Conversely, we did not find a significant difference in KOR function between late AIE- and water-exposed female rats, suggesting that the lower dopamine release observed in late AIE-exposed females is likely driven by a mechanism not dependent on KORs. Nevertheless, our data show that early AIE exposure in females resulted in augmented KOR function, as shown by greater inhibition of dopamine release when compared to water-exposed controls. This effect was not observed in early AIE-exposed males.

Ontogenetically, mRNA for KORs has been observed as early as gestational day (G) 12–PD 9, and the system seems to be fully matured by PD 21–25 [55]. Most studies show that the KOR mRNA expression and binding increases or fluctuates up to PD 9–10, followed by stable levels until PD 22–40 [56,57]. One study observed KOR mRNA expression levels to be significantly lower at PD 5 compared to adulthood [58]. It is important to note, however, that the inhibitory effect of KORs on dopamine release in the NAc decreased from PD 7 to PD 21 [59]. These studies, using male rats, suggest that the KOR system matures by early adolescence. The KOR system development in females is largely understudied, thus comparable information in females is unknown. It is possible that the sex and exposure age differences that we observe are likely driven by the interaction between KORs and their ability to inhibit dopamine release, and that AIE exposure at distinct time points (early vs. late adolescence) differentially affects KOR control over dopamine release. More research in this area is necessary to fully understand the underlying mechanisms.

Ethanol exposure during various developmental stages also differentially impacts KOR mRNA and function. Studies have shown that repeated prenatal ethanol exposure between G 17–20 results in higher levels of KOR mRNA in the NAc on PD 8 [60]. In contrast, similar prenatal ethanol exposure decreased KOR protein levels in the NAc on PD 14–15 [61]. Studies in neonatal rats have shown that activation of KORs is necessary to produce behavioral reinforcing effects of ethanol [62,63]. In addition, KOR inhibition blocked operant responding to ethanol in pre-weanling rats assessed on PD 14–17 [64]. Together, these studies suggest that the relationship between ethanol and KORs is bidirectional—that is, ethanol exposure affects KOR function, KOR mRNA, and protein expression. In turn, KOR function contributes to the behavioral effects of ethanol. These effects, however, are age- and sex-dependent. While inhibition of KORs in non-dependent adult male rats promoted ethanol intake, no changes were observed in adolescent (PD 25–38) rats. In contrast, KOR inhibition reduced ethanol intake in adult females, with no changes evident in their adolescent counterparts [36]. Although these data suggest that KORs likely do not play a major role in adolescent ethanol consumption, it is important to note that ethanol exposure used in this study was mild, and KOR inhibition effects on drinking were examined in non-dependent rats. Repeated exposure to ethanol, as used in the prenatal experiments discussed earlier, may be necessary to induce long-term changes in KOR function across the two sexes.

While prenatal ethanol exposure did not result in sex differences [60,61], it is possible that ethanol exposure during the peri-pubertal period may affect the dynorphin/KOR system in adulthood. Secretion of dynorphin in the hypothalamus decreases at the onset of puberty in female rats [65]. Alcohol exposure between PD 27 and 33 in female rats, however, results in elevated synthesis of dynorphin in this region [66]. Thus, it is possible that exposure to ethanol around the onset of puberty in females (PD 30–32) [67] results in retention of adolescent-typical enhancement in KOR function evident in the early-AIE-exposure female cohort in the current study. The KOR system is likely arrested in this altered state as a result of excessive ethanol exposure, even after the termination of ethanol administration. Because late-AIE exposure occurred after puberty in the late-AIE exposed females, this effect was not observed in this group. It is possible that because early-AIE exposure in males ends right around the onset of puberty (PD 40–44) [67], with very few exposures occurring during this time, dynorphin levels might not be influenced, resulting in typical KOR function in adulthood. The augmented KOR function observed following late-AIE exposure in males is likely not related to puberty, as most exposures occurred after sexual maturation. Further studies in adolescent males and females are needed for investigation of adolescent-typical KOR function before and during pubertal maturation, given that the role of puberty in KOR function is still not well documented or understood.

KOR function can also be altered as a result of changes in efficacy or potency. Our data showed that the augmented KOR function in early-AIE-exposed females was a result of augmented efficacy, as shown by the greater maximum effect of KOR activation on the inhibition of dopamine release, as well as potency, as shown by lower EC50 of U50,488. On the other hand, the attenuated KOR function observed in late AIE-exposed males was driven by a reduction in potency of the receptor. These data suggest that these KOR changes in males and females are mechanistically distinct.

The relationship between dopamine kinetics and KOR function is complex. Repeated KOR activation [68], CIE exposure [21,22], and adolescent adverse exposure, such as social deprivation [32,69], increase aversive behavioral responses and cocaine place preference [68], as well as anxiety-like behaviors and ethanol consumption [21,22,32,69]. These effects are likely associated with hyperfunctioning KORs, as the inhibition of KORs blocks KOR-activation-mediated, cocaine-conditioned place preference, reverses anxiety-like behaviors, and reduces ethanol consumption. KOR hyperactivity leads to enhanced inhibition of stimulated dopamine release, as well as an attenuation in tonic extracellular levels of dopamine [32]. Changes in KOR function are not expected to have an impact on stimulated dopamine release at baseline in an ex vivo preparation (e.g., ex vivo FSCV used in the current study), as there is no evidence of dynorphin tone in a slice. However, activation of KORs by exogenous application of the agonist U50,488 does in fact reduce dopamine release, as reported by previous studies [22,32,33]. It is possible that dopamine transmission changes may affect the function or expression of membrane-bound or active KORs. For example, one study has shown that lesioning mesolimbic dopamine neurons in the NAc shell leads to a reduction of dynorphin synthesis (measured by diminished mRNA levels; [70]). An attenuation in ligand synthesis may in fact alter receptor function; however, the directionality of this change could be in either direction. Thus, overall it is possible that initial changes in dopamine transmission may affect KOR function, or that initial changes in KOR function may affect dopamine transmission. A more systemic approach is necessary to tease these differences apart.

Based on previous behavioral findings showing no effects of late-AIE exposure on anxiety-like behavior and social interaction in females [12,13], we did not predict any changes in dopamine transmission or KOR function. Therefore, the attenuation of stimulated dopamine release was surprising. It is possible that the prolonged abstinence period leads to neuroadaptations that drive lower stimulated pre-synaptic dopamine release and an overcompensation of post-synaptic receptor changes, resulting in typical behavioral outcomes. Thus, the behavioral outcomes observed in prior studies may be, at least in part, driven by post-synaptic receptor changes. The heightened anxiety-like behavior evident in late-AIE-exposed male rats [12] can be attributed to the greater stimulated dopamine release in the NAc core observed in the current study. Previous studies have linked enhanced stimulated dopamine release in the NAc core to augmented anxiety-like behaviors in male rats [32,71]. Early-AIE-exposed male and female rats exhibit heightened anxiety-like behaviors [12], and attenuated social behaviors in males [13]; however, we did not observe any early-AIE-associated alterations in dopamine transmission in either sex. It is important to note that the dopamine system control over social behaviors is attributed to post-synaptic D1 receptors in the NAc [14]. Thus, it is possible that the behavioral outcomes reported in previous studies are due to a combination of pre- and post-synaptic changes in dopamine signaling or isolated to por-synaptic changes only. Moreover, developmental maturity of the system that varies between sexes, and time/age of ethanol exposure may contribute to these changes.

In humans, alcohol use during adolescence increases the risk of developing AUD in adulthood; interestingly, this occurs in an age- and sex-dependent manner. For example, an earlier age of onset (during adolescence) increases the risk of AUD in females, while late-adolescent age of onset increases risk of AUD in males [72]. In the current study, we showed dysregulation of KOR function in early-AIE-exposed females and late-AIE-exposed males. Though these changes are in opposite directions, augmentation in females and attenuation in males, the age- and sex-specific disruption of normalcy roughly matches the behavioral outcomes in humans. A recent study sampling an urban male population between ages 12 and 22 showed that underage drinking produced hyperactivity in the ventral striatum in response to reward anticipation [73]. Interestingly, the ventral striatum volume was observed to be smaller in alcohol binge drinkers compared to alcohol-naïve males, and greater in alcohol binge drinkers compared to alcohol-naïve females [74]. We do not know whether these volumetric changes were specific to a neuronal system (e.g., GABAergic, glutamatergic, dopaminergic, etc.), but it is interesting that the observed volumetric changes were opposite in males and females; similar to opposing changes in stimulated dopamine release in late-AIE-exposed males and females in the current study.

In summary, the results of the current study showed that while early AIE exposure followed by protracted abstinence did not affect dopamine kinetics in male or female rats, KOR-activation mediated inhibition of dopamine release was observed to be augmented in early-AIE-exposed females only—an effect driven by enhanced potency and efficacy of the receptor. On the contrary, late-AIE exposure followed by protracted abstinence resulted in the facilitation of stimulated dopamine release in males and attenuation in females at baseline. Moreover, we observed a reduction in KOR-activation mediated inhibition of dopamine release, driven by selectively enhanced potency in males. Though we did not test direct mechanisms in this study, it is possible that the facilitation in stimulated dopamine release was driven by reduced KOR function in late-AIE-exposed male rats. This relationship was not observed in early or late AIE-exposed females. Together, these data suggest that the impact of AIE on dopamine transmission and KOR function is exposure age- and sex-dependent, suggesting that AIE-associated changes in males and females may be driven by distinct mechanisms. Interestingly however, the age- and sex-dependent dimorphic changes observed in the current study map onto age- and sex-dependent changes observed in the human literature. These changes could not only affect alcohol consumption in adulthood, but could also affect reward processing, thus increasing pro-addictive behaviors. Further research is necessary to elucidate the exact mechanisms that drive these neural adaptations.
